# Use of the Intratumoural Anticancer Drug Tigilanol Tiglate in Two Horses

**DOI:** 10.3389/fvets.2020.00639

**Published:** 2020-09-09

**Authors:** Thomas De Ridder, Mick Ruppin, Meagan Wheeless, Stephanie Williams, Paul Reddell

**Affiliations:** ^1^QBiotics Group Ltd., Yungaburra, QLD, Australia; ^2^Tableland Veterinary Service, Malanda, QLD, Australia; ^3^Tableland Veterinary Service, Edmonton, QLD, Australia

**Keywords:** equine, sarcoid, carcinoma, ocular, neoplasia, intratumoural, protocol

## Abstract

Tigilanol tiglate is a novel small molecule approved as a veterinary pharmaceutical in Europe for intratumoural treatment of non-metastatic, non-resectable canine mast cell tumors. The drug has a “tumor agnostic” mode of action associated with induction of an acute inflammatory response at the treatment site, immune cell recruitment, and disruption of tumor vasculature. Consequently, tigilanol tiglate has potential in treating a range of tumor types in humans and companion animals. However, it is likely that species-specific dosing and concomitant medication protocols will be required, especially to manage the drug-induced acute inflammatory response at the treatment site. As an initial step in evaluating tigilanol tiglate for treating cutaneous tumors in horses, we developed an equine-specific protocol involving (a) a 30% reduction in intratumoural tigilanol tiglate dose rate compared to that used in dogs, and (b) a regime of concomitant medications to manage the drug-induced acute inflammatory response at the treatment site. Here we report a preliminary study in two horses using the protocol to treat (i) an aggressive fibroblastic sarcoid that had recurred following surgical excision and (ii) a fast-growing peri-ocular squamous cell carcinoma. Clinical response to tigilanol tiglate treatment in these cases was similar to that observed in canine and human patients. Localized inflammation and bruising developed rapidly at the treatment site with haemorrhagic necrosis of the tumor evident within 24 h. Slough of necrotic tumor mass occurred within 6–16 days followed by infill of the tissue defect and full re-epithelialisation of the treatment site with good functional outcome. Drug-induced inflammation and oedema at the treatment site were well controlled by the concomitant medications and largely resolved within 3 days, while the wound that formed following tumor slough healed uneventfully. Both patients displayed minor lethargy during the first 36 h after treatment and localized treatment-site discomfort was apparent over the first 3–5 days. There was no evidence of recurrence of the sarcoid at 93 days, or the squamous cell carcinoma at 189 days. The results from this study support continued development and evaluation of tigilanol tiglate as a potential future treatment option for cutaneous equine tumors.

## Introduction

Tigilanol tiglate (also known as EBC-46) is a novel small molecule approved in the European Union and United Kingdom as an intratumourally-administered, veterinary pharmaceutical for treatment of non-metastatic, non-resectable canine mast cell tumors (1). It is also under clinical evaluation as an intratumoural treatment for a range of other cutaneous and subcutaneous cancers in humans and companion animals (2–4). Tigilanol tiglate is a potent cellular signaling molecule with a multifactorial mode of action that induces (a) a rapid, acute and highly localized inflammatory response in and immediately surrounding the tumor mass, (b) recruitment of immune cells, (c) loss of tumor vasculature integrity and (d) induction of tumor cell death by oncosis (5). At efficacious doses, these processes lead to haemorrhagic necrosis and destruction of the tumor within 7 days followed by resolution of the resulting wound (tissue defect) with good functional and cosmetic outcomes (1). Because this multifactorial mode of action relies in significant part on treatment response by “host” tissues (e.g., immune cell recruitment and effects on tumor vasculature), rather than intrinsic sensitivity of the tumor cells *per se*, tigilanol tiglate can be considered “tumor agnostic” and has potential for efficacy against a range of different tumor types (irrespective of cells of origin or specific cancer gene mutations) across different animal species.

Sarcoids and squamous cell carcinomas (SCCs) are common skin tumors in horses (6, 7). While they rarely metastasise, both tumor types can be highly invasive into local tissue and consequently they can be difficult to treat effectively with current standards of care (8, 9), particularly when in proximity to vital tissues such as on the head, perineal region and limb joints. They also have relatively high recurrence rates and, in the case of sarcoids, a tendency to become refractory to subsequent interventions (6).

As the first stage of a research program to evaluate the potential of tigilanol tiglate for treatment of cutaneous equine tumors, a pilot safety study of tigilanol tiglate was conducted in horses. That unpublished study showed that when tigilanol tiglate was injected subcutaneously into the normal equine skin, the resulting drug-induced acute inflammatory response at the treatment site was more robust and caused more extensive local oedema than observed in canines treated subcutaneously with similar doses. This observation is consistent with (a) the recognized greater sensitivity of horses to many inflammatory challenges (e.g., as reflected in over exuberant responses that predispose them to common inflammatory conditions such as asthma, colic and laminitis) and (b) the respective differences between horses and dogs in the nature and initiation of their acute phase response to trauma (10, 11).

Based on these clinical observations, it was considered that current tigilanol tiglate protocols for treatment of neoplasia in dogs and humans may be inappropriate for horses. In particular, the development of more excessive local oedema associated with the acute drug-induced inflammatory response at the treatment site could result in more extensive wounds following tumor slough. To address this, an equine-specific tigilanol tiglate treatment protocol aimed at reducing the extent of the acute inflammatory response that may occur at the treatment site is under preliminary investigation. This protocol involves a combination of (a) standing sedation, (b) reduced tigilanol tiglate dose rate, and (c) a regime of concomitant anti-inflammatory medications following treatment.

Here we report two initial equine cases treated using this equine-specific tigilanol tiglate protocol. One example is of an aggressive fibroblastic sarcoid that had recurred following previous surgical resection, the second involved a rapidly growing SCC in the left medial canthus where additional measures were used to protect and minimize local inflammatory effects on the eye.

## Methods

QBiotics Group was approached by attending veterinarians to enquire if two difficult cases in their care could be considered for treatment as preliminary studies for evaluating the safety and efficacy of tigilanol tiglate treatment for equine tumors. It was subsequently agreed to treat both cases under a study protocol covered by animal ethics on the basis that (i) the tumors were of modest size (<6 cm^3^ in volume) requiring a conservative dose of <2 mg tigilanol tiglate, (ii) the two horses were generally healthy other than the presenting lesion, and (iii) available standards of care offered (surgery or conventional chemotherapy with injectable cisplatin) were likely problematic in resolving the presenting lesions and were declined by the owners. Prior to treatment the owners received information about tigilanol tiglate and were reminded of other available treatment options before signing treatment consent forms. Brief details of the presentation and case history of each patient are summarized below.

### Case Histories

#### Fibroblastic Sarcoid That Recurred After Previous Surgical Resection

**Figure 1:**
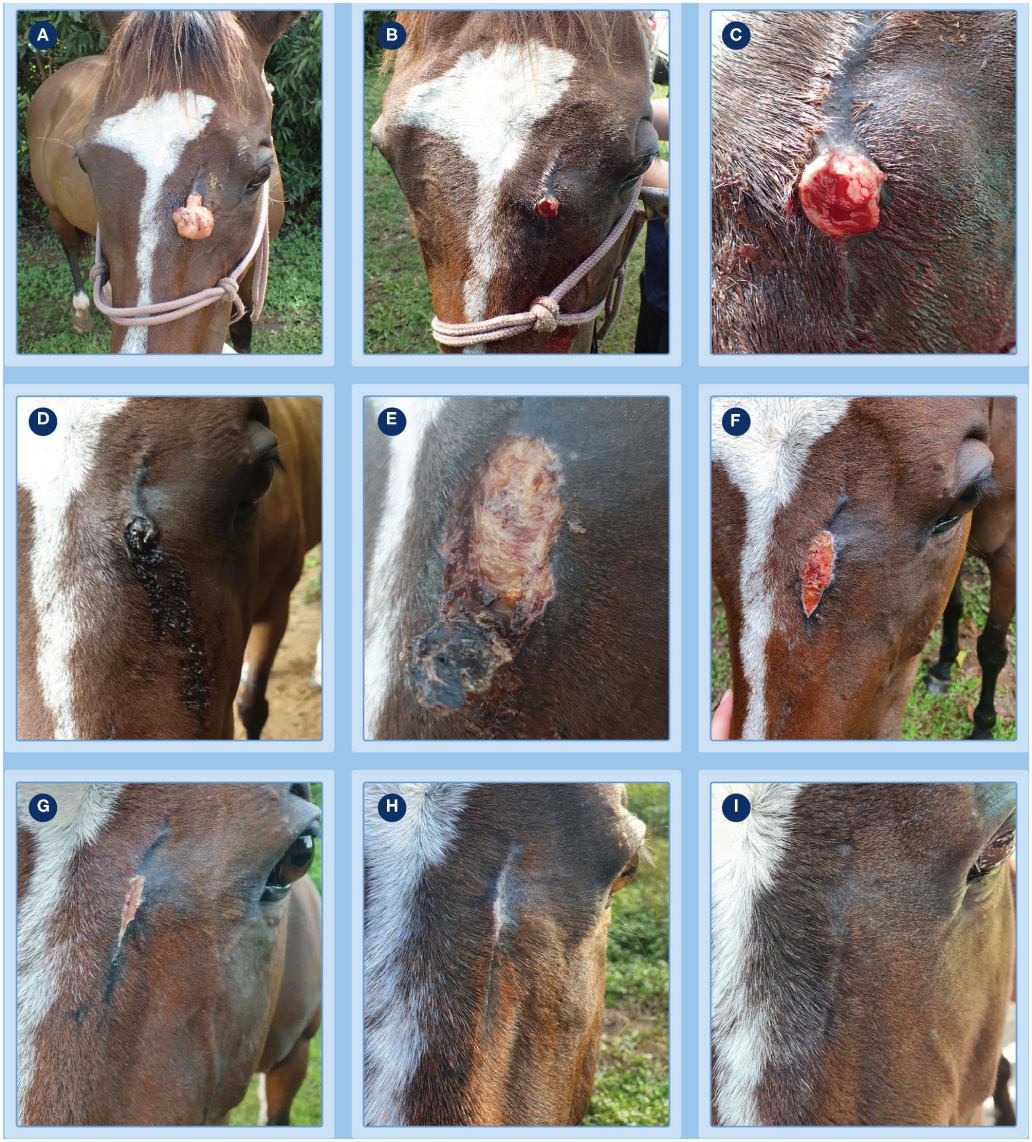
Progression of clinical response of a fibroblastic sarcoids to treatment with tigilanol tiglate. **(A)** the pedunculate sarcoid prior to treatment; **(B,C)** treatment site after pedicle had been surgically excised to allow measurement of sarcoid base for calculation of dosing and immediately prior to injection with tigilanol tiglate **(D)** the development of bruising and haemorrhagic necrosis by 24 h after injection; **(E)** tumor slough at day 6; **(F–H)** progression of healing at days 15, 27, and 36; **(I)** no evidence of sarcoid recurrence at the treatment site by day 93.

An 11-year old Thoroughbred mare presented to Tableland Veterinary Service in February 2020 with an aggressive pedunculate fibroblastic sarcoid on the forehead medial to the left eye (Figure 1A) that had regrown following *en bloc* surgical resection with margins in late October 2019. At the time of the original surgery, the presenting sarcoid had been diagnosed as verrucous by the attending veterinarian based on its clinical appearance and behavior.

#### Peri-ocular SCC

**Figure 2:**
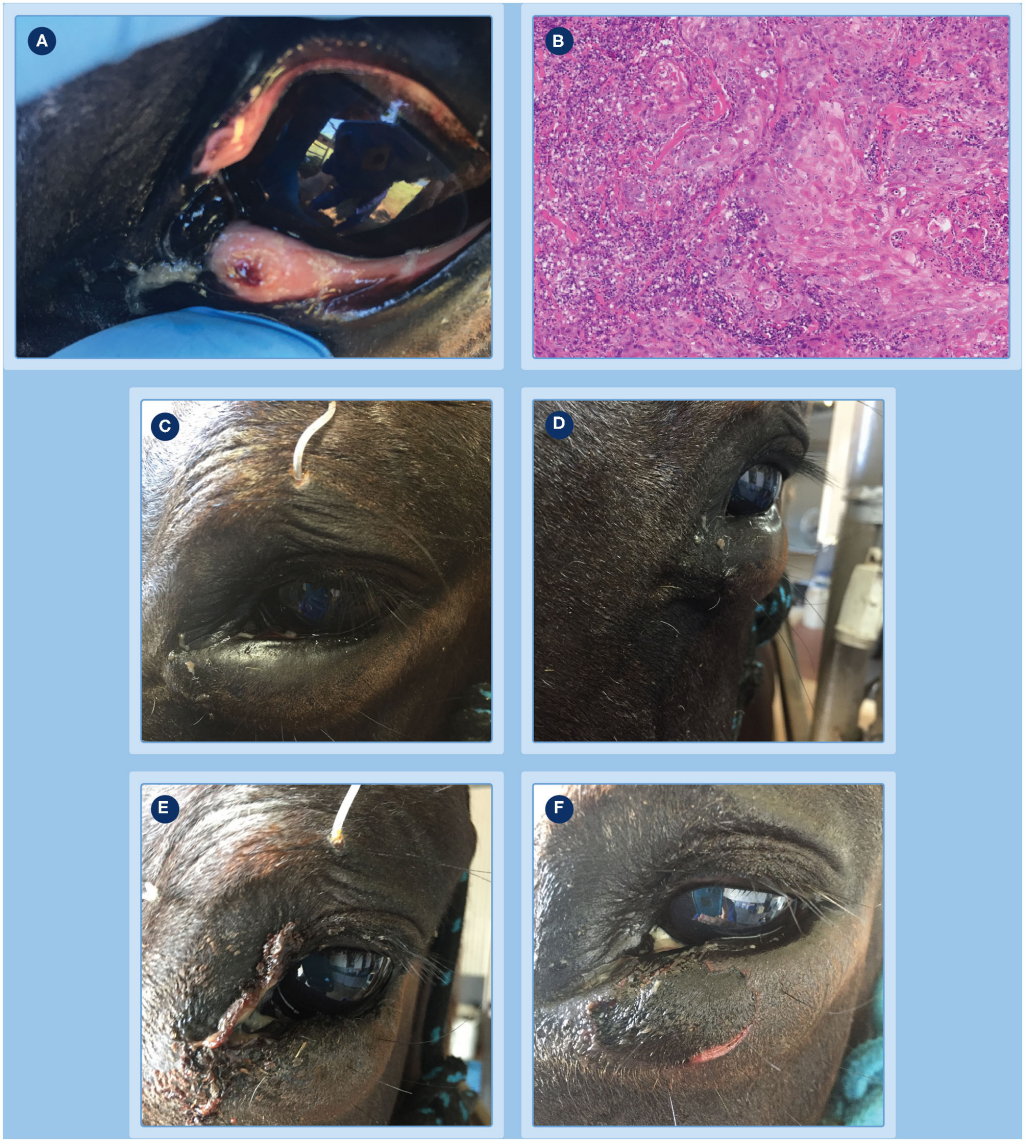
Progression of clinical response of a peri-ocular SCC to treatment with tigilanol tiglate. **(A)** the tumor pre-treatment; **(B)** histopathology confirming the diagnosis as an SCC; **(C,D)** the extent of local swelling and inflammation that had developed by 24 h; **(E)** haemorrhagic necrosis of the tumor and associated discharge of exudate at day 3; **(F)** markedly circumscribed and blackened area at the treatment site with eschar formation becoming visible at day 4.

A 22-year old Thoroughbred x Clydesdale gelding presented to the referring veterinarian in early October 2019 with a 4 mm ulcerated and rapidly growing lesion of the left medial canthus (Figure 2A). Ten months previously, the patient's right eye had been enucleated (mid December 2018) after histological diagnosis of a SCC in the right medial canthus. Due to the location and patient history, it was suspected that the new lesion was likely an SCC.

### Treatment Protocol

The tigilanol tiglate treatment protocol used in these two preliminary studies was based on an analysis of the results of the pilot safety study in horses and focused on (a) ease of drug administration and (b) minimizing the treatment site inflammatory response induced by the drug without potentially compromising treatment efficacy. There were six components to the protocol: (i) standing sedation, (ii) local or regional anesthesia, (iii) estimation of tumor volume, (iv) calculation of tigilanol tiglate dose, (v) dose administration, and (vi) concomitant medications to manage the local acute inflammatory response (see Table 1 for details). For lesions in the muco-cutaneous and peri-orbital tissues near the eye, additional interventions involving placement of an ocular lavage treatment tube to deliver ocular medications were included in the protocol to manage local inflammation and prevent potential development of uveitis (Table 1).

**Table 1:** Overview of tigilanol tiglate treatment protocol under development for equine sarcoids and squamous cell carcinomas, including ocular and peri-ocular lesions.

**Protocol component**	**Description/Comments**
**1. Sedation**	
	• Standing sedation at veterinary discretion. Routine sedation used in cases includes 0.1 mL/100 kg of detomidine hydrochloride (10 mg/mL) and may include 0.1 mL/100 kg of butorphanol (10 mg/mL).
**2. Local or regional anesthesia**	
*2a. All patients*	• May be required to minimize patient movement to facilitate adequate infiltration into the sarcoid mass, depending on location (e.g., pinnae) and/or size. • Used also to facilitate post-treatment biopsies where appropriate.
*2b. Ocular/peri-ocular lesions*	• Auriculopalpebral nerve block to minimize patient movement and facilitate adequate infiltration into the tumor mass. • Local anesthesia of the eye can be increased with eye drops such as Proxymetacaine hydrochloride 0.5%. • Used also to facilitate post-treatment biopsies where appropriate.
**3. Estimation of tumor volume**	
	• Tumor volume calculated using caliper measures and based on a modified formula for an ellipse where T_volume_ (cm^3^) = ½ (lesion length x lesion width × lesion thickness).
**4. Calculation of tigilanol tiglate dose**	
*4a. General*	• Dose rate: 0.35 mg tigilanol tiglate (1 mg/mL) per cm^3^ of estimated tumor volume. • Minimum dose: 0.1 mg for small tumors ≤ 0.3 cm^3^ in volume. • Maximum dose per treatment: 2 mg per animal, equivalent to ≤ 6 cm^3^ total tumor volume per animal.
*4b. Ocular mucocutaneous lesions*	• Minimum dose: 0.05 mg of 1 mg/mL tigilanol tiglate. • Maximum dose per treatment: 0.2 mg of 1 mg/mL tigilanol tiglate.
*4c. Peri-orbital lesions*	• Minimum dose: 0.05 mg of 1 mg/mL tigilanol tiglate. • Maximum dose per treatment: 2 mg and tumors of ≤ 6cm^3^.
**5. Dose administration**	
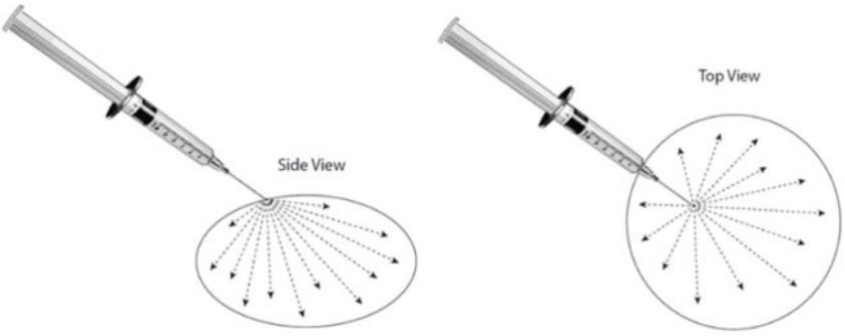	• Injected intratumourally using a single injection site where possible to reduce potential leakage of the dose. • Delivered using 1 mL Luer lock syringe with 26G ¾” needle. • Administered using intratumoural “fanning” technique (as illustrated on the left) to achieve maximum distribution and perfusion throughout the tumor.
**6. Concomitant medications**	
*6a. Immediately following treatment*	• Single dose of flunixin meglumine (50 mg/mL) administered IV *via* the jugular vein at the standard dose of 1.1 mg/kg.
*6b. Post-treatment*	• Phenylbutazone at 4.4 mg/kg BID administered orally for at least the first 3 days, can be reduced to 2.2 mg/kg BID for the next 7 days if required.
*6c. Ocular mucocutaneous and peri-orbital lesions **(critically important)***	• Ocular palpebral lavage treatment tube to be placed at time of treatment. • Ocular NSAID such as ketorolac trometamol 0.5% or similar, 0.1 mL q6 h until tumor slough. • Ocular atropine sulfate monohydrate 1% drops, 0.1 mL q6 h until tumor slough. • UV excluding head veil. • Monitor closely until tumor sloughs.

Treatments to both patients were administered by the referring veterinarians following the protocol and under guidance from a QBiotics veterinarian. The sarcoids patient was treated in the field in the owner's agistment paddock, while the SCC patient was admitted to the referring equine hospital for treatment and boarding to monitor treatment response over the first 14 days (note that boarding and monitoring was continued until day 20 as the owner was on holidays). Following sedation and local anesthesia (if required), the calculated dose of tigilanol tiglate (1 mg/mL in 40% propylene glycol) was administered with a 1 mL Luer lock syringe fitted with a 26G ¾” needle using an intratumoural “fanning” technique to achieve maximum distribution and perfusion of the tigilanol tiglate throughout the lesion (see illustration in Table 1). In the case of the sarcoids patient, following sedation the pedicle of the lesion was excised with a scalpel so that the remaining base of the tumor could be measured to estimate tumor volume and subsequently treated (Figures 1B,C). Specifics of sedation, local anesthesia, estimated tumor volume, calculated dose of tigilanol tiglate, and anti-inflammatory concomitant medications for the two patients are summarized in Table 2.

**Table 2:** Specific details of treatment protocol for the two equine cases treated with intratumoural tigilanol tiglate.

**Protocol component**	**Patient**	
	**Fibroblastic sarcoid**	**Peri-ocular SCC**
**Sedation**		
	0.5 mL detomidine hydrochloride (Dozadine injection 10 mg/mL; Virbac, Australia).	0.5 mL detomidine hydrochloride (Dozadine injection 10 mg/mL; Virbac, Australia) and 0.5 mL butorphanol (Butorgesic 10 mg/mL; Ilium Troy, Australia).
**Local anesthesia**		
	Nil, patient judged to be well sedated with good restraint to allow adequate drug infiltration into the tumor without leakage.	Auriculopalpebral nerve block using mepivacaine hydrochloride (Mepivacaine injection 20 mg/mL; Nature Vet, Australia). Additional ocular local anesthetic drops added throughout procedure as needed.
**Other interventions prior to tigilanol tiglate treatment**		
	Once sedation had taken effect, the pedicle of the pedunculated mass was excised with a scalpel (Figures 1A–C) so that the base of the tumor could be measured to estimate tumor volume. Excised tissue was retained for histopathology.	• Proxymetacaine hydrochloride 0.5% (Alcaine 0.5%) eye drops applied to the globe and conjunctival tissue. • Ocular lavage treatment tube placed in upper eyelid to facilitate delivery of ocular medications post tigilanol tiglate treatment.
**Estimated tumor volume (cm**^**3**^**)**		
	1.4	0.4
**Tigilanol tiglate dose (mL)**		
	0.5 mL	0.15 mL
**Concomitant medications**		
a. Immediately following treatment	10 mL flunixin meglumine (Flunixon 50 mg/mL; Norbrook, UK) - IV *via* the jugular vein.	10 mL flunixin meglumine (Flunixon 50 mg/mL; Norbrook, UK) - IV *via* the jugular vein.
b. Post-treatment	Oral phenylbutazone granules (Butalone Granules 1 g; Apex Laboratories, Australia) 4.4 mg/kg BID for first 3 days, reduced to 2.2 mg/g BID for a further 7 days.	Phenylbutazone paste (Bute Paste 200 mg/mL, Ranvet, Australia) 5 mL PO BID for first 14 days, reduced to 5 mL PO SID until 20 days^*^ post treatment. Light excluding head veil to minimize UV exposure to treated eye for 20 days^*^ post treatment.
c. Management of ocular inflammation	Not applicable	0.1 mL of ketorolac trometamol 0.5% (Acular 0.5%, Allergan, Australia) and 0.1 mL of atropine sulfate monohydrate (Atropine 1.0% eye drops, Minims, Australia) delivered *via* the ocular lavage tube every 6 h until tumor slough (day 16).

* *Standard protocol duration for these treatments was until tumor slough (in this case day 16). However, the patient remained in boarding because the owner was on holidays and these treatments were maintained over this additional period*.

Tissue samples were taken from the lesions of both patients for histopathological confirmation of the disease. In the case of the SCC patient, a biopsy was taken 14 days prior to tigilanol tiglate treatment, while for the sarcoids patient the pedicle that was excised immediately prior to administration of tigilanol tiglate was used.

### Assessment of Treatment Response

Treatment response was assessed from a combination of clinical observations made by veterinarians and the owners, and from sequential digital photographs of the treatment site in the days following treatment. Clinical observations included overall patient health and demeanor, extent of inflammation/oedema, evidence of local pain and/or discomfort, time for tumor slough, the condition and size of the resulting wound, and the progress of wound healing. In the case of the SCC patient boarded at the equine hospital, more detailed daily monitoring included checks of temperature, pulse rate and respiratory rate (TPR), and observation of the treatment site and eye for specific signs of hypopyon, uveitis, and blepharospasm. Both patients were also monitored over the course of the study for evidence of development of any new tumors at other sites on the body.

Treatment was considered successful if the tumor had completely resolved and there was full re-epithelialisation of treatment site wound (tissue defect) resulting from tumor destruction. The longer-term durability of this response continues to be monitored every 3 months using digital images, veterinary examination, and reports from the owners.

## Results

Following treatment with tigilanol tiglate, the tumors in both equine patients followed a pattern of clinical response consistent with that seen and reported with the drug in other species and tumor types including canine mast cell tumors (1, 3), a variety of human neoplasias (4), and murine tumors (2, 5). This clinical response is directly related to the mode of action of tigilanol tiglate in tumor destruction and involves localized bruising and inflammation/oedema developing at the treatment site within the first 24 h, followed by haemorrhagic necrosis of the tumor mass and finally slough of the necrotic tumor leaving a treatment site wound which usually heals uneventfully *via* secondary intention without the need for bandaging or other interventions (1).

### Fibroblastic Sarcoid

The progression of clinical response of the fibroblastic sarcoid to treatment with tigilanol tiglate is shown in Figure 1. There was clear evidence of bruising, slight oedema and haemorrhagic necrosis of the lesion within 24 h of treatment (Figure 1D) followed by sloughing of the necrotic sarcoid mass by day 6 (Figure 1E). Healing of the treatment site was well progressed by day 15 (Figure 1F) with full re-epithelialisation occurring within 36 days after treatment (Figures 1G,H). There was no recurrence of the treated sarcoid 93 days after treatment (Figure 1I) and no sarcoids had developed elsewhere on the patient during this period. Other than discomfort at the treatment site, mild lethargy and reduced feed intake over the first 36 h, there were no other signs of changes in patient behavior or demeanor recorded by the referring veterinarian or owner following treatment.

### Peri-ocular/Mucocutaneous SCC

**Figure 3:**
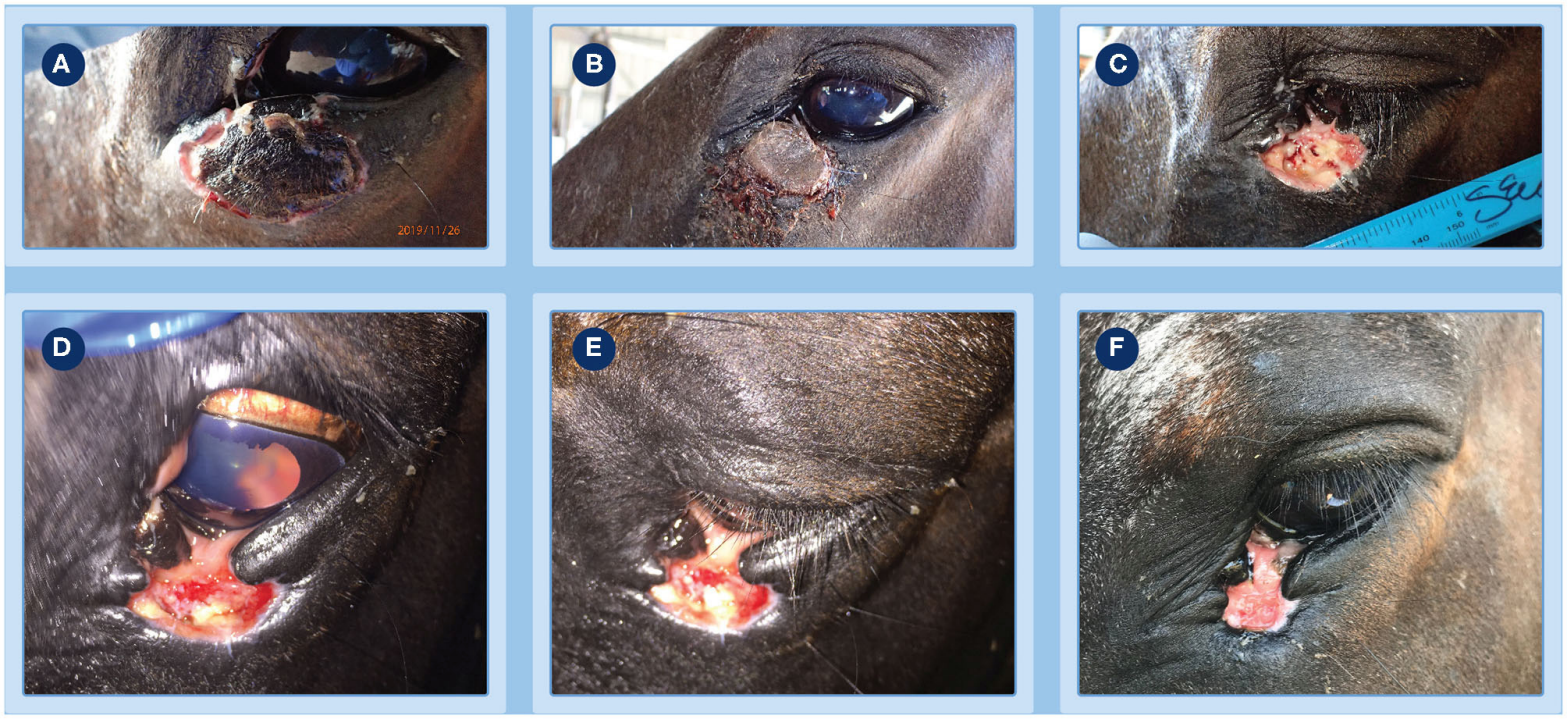
Progression of clinical response of a peri-ocular SCC to treatment with tigilanol tiglate. **(A,B)** clear demarcation of the necrotic tumor mass at days 8 and 16, respectively; **(C)** underlying wound bed exposed following slight manual pressure to the eschar at day 16; **(D,E)** examination of the eye at day 19 show it to be normal and unaffected with a well-dilated pupil; **(F)** healing at the site by day 27.

**Figure 4:**
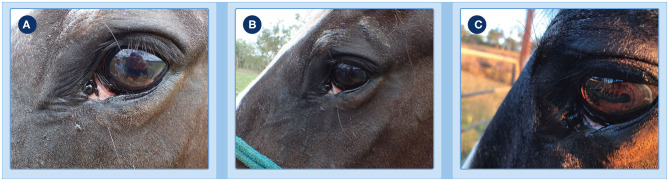
Progression of clinical response of a peri-ocular SCC to treatment with tigilanol tiglate. Healed treatment site at day 73 **(A,B)** and day 189 **(C)** showing no recurrence of the tumor.

The progression of clinical response of the peri-ocular SCC to treatment with tigilanol tiglate is shown in Figures 2–4. Figure 2A shows the SCC prior to treatment, while Figure 2B is the histological section taken 2 weeks prior to treatment confirming a squamous cell carcinoma. Within 24 h of treatment, swelling and inflammation at, and immediately surrounding, the treatment site was clearly evident (Figures 2C,D); also note the placement of the ocular lavage treatment tube in the top eyelid in Figure 2C. Haemorrhagic necrosis of the tumor mass had occurred by day 3 and was accompanied by a discharge of exudate (Figure 2E), with a marked circumscribed and blackened area with eschar formation becoming more visible by day 4 (Figure 2F).

Further demarcation of the necrotic tumor mass developed over the next 4 days (Figure 3A, day 8). At day 16, after light sedation and slight manual pressure to the eschar site, the mass completely sloughed (Figures 3B,C). Granulation tissue rapidly infilled the exposed wound over the next 3 days and by day 19 the wound had reduced in size (Figures 3D,E) compared to day 16 (Figure 3C). Throughout this period, administration of ocular and oral NSAIDs, along with ocular atropine, were continued. Examination of the eye at day 19 showed it to be normal and unaffected with a well dilated pupil (Figure 3D) and while a small wedge of functional lower eyelid near the medial canthus had been lost following tumor destruction and slough, this did not affect the ability of the eye to blink. The patient was discharged from hospital the following day and the referring veterinarian was comfortable that no further medication was required. However, the owner was asked to monitor the patient and make contact if there was any evidence of obvious pain such as physically closing of the eye, as well as excessive lacrimation or blepharospasm that could indicate uveitis development or corneal ulceration. A routine follow-up assessment was made 7 days after discharge from hospital, the eye continued to be normal and the wound had further reduced in size (Figure 3F, day 27). There was no further veterinary follow-up until 73 days by which time the treatment site had completely healed (Figures 4A,B). At a routine assessment scheduled for ~6 months after treatment (day 189) there was no evidence of recurrence and the eye was fully functional (Figure 4C). At this time there was also no evidence of metastasis, either locally or to more distant sites on the patient.

Other than reduced feed intake over the first 24 h and discomfort at the treatment site associated with the inflammatory response over the first 7 days, the patient showed no other adverse clinical or behavior signs directly associated with the treatment. Regular daily cleaning around the necrosing tumor with gauze swabs soaked in 0.9% saline, the use of a head veil to exclude light, and administration of ocular medications was continued to minimize discomfort associated with the initial tissue loss of the medial canthus and eyelid functionality, and potential uveitis formation (though considered a remote chance of occurring after day 16) until the patient was discharged from boarding 20 days after treatment.

## Discussion

In this preliminary study we have shown that an equine-specific treatment protocol based on reduced intratumoural tigilanol tiglate dose and a regime of concomitant anti-inflammatory medications resulted in complete resolution, with good cosmetic and functional healing outcome at the treatment site, of (i) a fibroblastic sarcoid that had recurred following surgical excision 4 months earlier and (ii) a fast growing peri-ocular SCC.

Our primary purpose in developing this protocol had been concern that horses were likely more sensitive to the acute inflammatory response induced by tigilanol tiglate at the treatment site than other species tested. This inflammatory response, mediated *via* cytokine and chemokine signaling, is an important component of the drug's multifactorial mode of action and functions to isolate the tumor mass (12) and stimulate recruitment of immune cells (5). However, if this inflammatory response is overly robust or persists longer than required for the drug's efficacy, it has potential to cause more extensive local oedema and result in the formation of larger wounds at the treatment site following tumor slough.

The results reported here show that our equine-specific protocol has been effective in these two initial cases in managing the drug-induced inflammatory response at the treatment site, and resulting wound size, through a combination of:

- A 30% lower intratumoural dose rate (0.35 mg drug/cm^3^ tumor volume) compared to that required for efficacious treatment of canine mast cell tumors (1, 3) and of a range of neoplasia in humans (4); and,
- A regime of concomitant non-steroidal anti-inflammatory medications (NSAIDs) administered at, and in the days following, treatment.

In respect to dose rate, it is interesting that despite the 30% lower tigilanol tiglate dose per unit of tumor volume, the progression and timing of critical clinical events associated with the mode of action of tigilanol tiglate in tumor destruction and subsequent wound resolution (*viz*. induction of the acute inflammatory response, haemorrhagic necrosis of the tumor, and tumor slough) in these equine cases was very similar to that reported previously in treatment of canine and human tumors (1, 4). This is consistent with horses having a higher sensitivity to inflammatory challenges and apparently lower thresholds, compared to canines and humans, for activation of key pro-inflammatory mediators (such as IL-1β and TNF-α) that are induced by tigilanol tiglate and which are involved in early stage initiation and amplification of acute phase responses (10, 11). It also provides initial evidence to suggest that the transient pro-inflammatory signaling cascades induced by, and contributing to the efficacy of tigilanol tiglate, are achieved at lower intratumoural concentrations of the drug in horses than in dogs and humans.

The regime of anti-inflammatory medications was also likely an important component of the protocol for (a) controlling the propagation of the inflammatory response and development of oedema beyond the treatment site and its immediate surrounds, and (b) contributing to the more rapid resolution of the initial acute pro-inflammatory signaling cascade that had been induced by the drug. Both aspects likely contributed to minimizing the size of the wounds that formed following tumor slough. In the peri-ocular SCC case, the additional interventions to manage the local inflammatory response, including the placement of the ocular treatment tube, were particularly critical, not only to control wound size but also to reduce pain in this sensitive area and to minimize the significant risk of uveitis and potential total loss of function. It is likely that the success of the treatment in this case, in destroying the tumor and resulting in only a very minor tissue defect and the undisrupted function of the eye, was in major part due to these specific additional elements of the treatment regimen.

No significant adverse events were recorded by the attending veterinarians or owners following the tigilanol tiglate treatments. Wounds that formed at the treatment site following tumor slough were managed without intervention and healed uneventfully by secondary intention. Both patients displayed minor lethargy during the first 36 h after treatment while discomfort was apparent at the treatment site over the first 3–5 days and was almost certainly associated with the drug-induced localized inflammatory response.

Overall, using the equine-specific protocol outlined in this study we have shown that an intratumoural dose of tigilanol tiglate resulted in the complete resolution of the target tumor followed by full re-epithelialisation of the treatment site. While both patients continue to be monitored, the lack of recurrence of an aggressive fibroblastic sarcoid 3 months after treatment, and of a rapidly-growing peri-ocular SCC at 6 months after treatment, is encouraging and indicative of a likely enduring nature to the treatment response.

Clearly, the clinical significance and application of these results is limited in that only two horses were involved in this preliminary study. Further clinical studies based on this equine-specific protocol are planned in the near future with the aims of (a) refining dosing and administration strategies and (b) establishing the clinical efficacy and safety of tigilanol tiglate in a statistically-relevant, representative equine patient population. These planned clinical studies will provide a better understanding of the potential equine use of the drug and underpin possible future development as a safe and effective treatment option for cutaneous equine tumors.

## Data Availability Statement

De-identified patient data supporting the conclusions of this article will be made available by the authors on request from qualified veterinarians and researchers.

## Ethics Statement

The animal study was reviewed and approved by Queensland Department of Agriculture and Fisheries, Community Animal Ethics Committee (AEC). Written informed consent was obtained from the owners for the participation of their animals in this study.

## Author Contributions

TD and PR were responsible for protocol design, data compilation, and manuscript preparation. MR, MW, SW, and TD performed the clinical work and reviewed the manuscript. All authors contributed to the article and approved the submitted version.

## Conflict of Interest

MR and SW are directors, and MW is an associated veterinarian, with Tableland Veterinary Service. Tableland Veterinary Service received payments from QBiotics Group Limited to cover other costs (e.g., sedation, concomitant medications, follow up assessments) associated with these equine patients treated with tigilanol tiglate. TD and PR are employed by QBiotics Group Limited. QBiotics Group Limited own the intellectual property and patents associated with tigilanol tiglate.
